# The effect of female hormone in otosclerosis. A comparative study and speculation about their effect on the ossicular chain based on the clinical results

**DOI:** 10.1007/s00405-022-07295-w

**Published:** 2022-02-21

**Authors:** Giampietro Ricci, Valeria Gambacorta, Ruggero Lapenna, Antonio della Volpe, Ignazio La Mantia, Massimo Ralli, Arianna Di Stadio

**Affiliations:** 1 Otolaryngology Department, University of Perugia, Perugia, Italy; 2 Otolaryngology Department, Silvestrini Hospital, Perugia, Italy; 3 Otology and Cochlear Implant Unit, Santobono-Posilipon, Naples, Italy; 4 Department G.F Ingrassia, University of Catania, Catania, Italy; 5 Organ of Sense Department, University La Sapienza of Rome, Rome, Italy

**Keywords:** Otosclerosis, Gender differences, Air bone gap, Conductive hearing loss, Estrogen, Surgery, Outcome

## Abstract

**Purpose:**

This study aimed at identifying gender differences in the hearing thresholds in a sample of patients with otosclerosis before and after surgery to understand the impact of female hormones on auditory thresholds.

**Methods:**

This retrospective study analyzed 184 patients (123 women and 61 men) affected by otosclerosis. All the patients were affected by conductive hearing loss and treated by stapedoplasty. Auditory thresholds at the baseline (T0) and one month after surgery (T30) were collected. Air and bone thresholds and Air Bone Gap (ABG) were compared between females and males using one-way ANOVA.

**Results:**

Statistically significant differences were observed comparing the air threshold at T0 vs T30 both in women and men (*p* < 0.0001). No statistically significant differences were observed in the bone conduction thresholds before and after surgery. The comparison between females and males showed statistically significant differences both at T0 (*p* < 0.01) and T30 (*p* < 0.05) for air conduction thresholds and ABG at 4000 Hz.

**Conclusion:**

Although stapedoplasty reduced the difference between females and males in the air conduction thresholds and ABG, women showed better recovery of their middle ear function with better auditory thresholds and ABG. The female hormones might positively impact the ligaments of the incudostapedial joint improving chain flexibility. This benefit might explain the statistically significant difference observed in women at 4000 Hz before and after surgery.

## Introduction

Several authors [1, 2] have shown that females have better auditory function than men analyzing subjects with sensorineural hearing loss (SNHL) [3]. These differences can be related to the effects of female hormones (mainly estrogen) [4] on the inner ear [5, 6] and/or on the auditory system [7, 8] at birth. In addition, different exposures to sound during life [9] and/or different comorbidities between females and males [9] can determine the differences overserved in the auditory thresholds of adults and elderly subjects.

Delhez et al. tried to explain the protective effects of female hormone that can explain this clinical difference [4]. The authors studied the effects of these hormones at cellular level and speculated that their findings might explain what happens in the inner ear as follows: (i) the estrogens increase the expression of the anti-oxidant genes Superoxide Dismutase (SOD) and thereby reduce Reactive Oxygen Species (ROS); because the increase of ROS induces apoptosis in the hair cells (HC), their reduction might benefit the survival of HC; (ii) a direct upregulation of anti-apoptotic genes, such as Bcl2 and Bcl-xL, could have a role in the protection and survival of HC and spiral ganglions (SGN); (iii) the estrogen upregulates neuroglobin, a potent ROS scavenger that, having a vasorelaxant effect, might improve the perfusion of inner ear and vascular stria preserving the HC [10]; (iv) E2 regulates many ion channels, including K + channels, which are expressed in strial cells, and are crucial for endolymph composition and mechanic transduction; (v) estrogens could reduce cochlear inflammation by inhibiting NLRP3 expression or activating cochlear resident macrophage-like cells and the release of anti-inflammatory cytokines [11]. Despite speculative, these explanations seem quite reasonable to justify the better hearing capacity of females.

Questions remain about what happens in specific conditions, commonly known to be cause–effect linked to the female hormones and which determines a conductive hearing loss (CHL), such as otosclerosis. Might the female hormones be (partially) beneficial also in this setting?

**Fig. 1:**
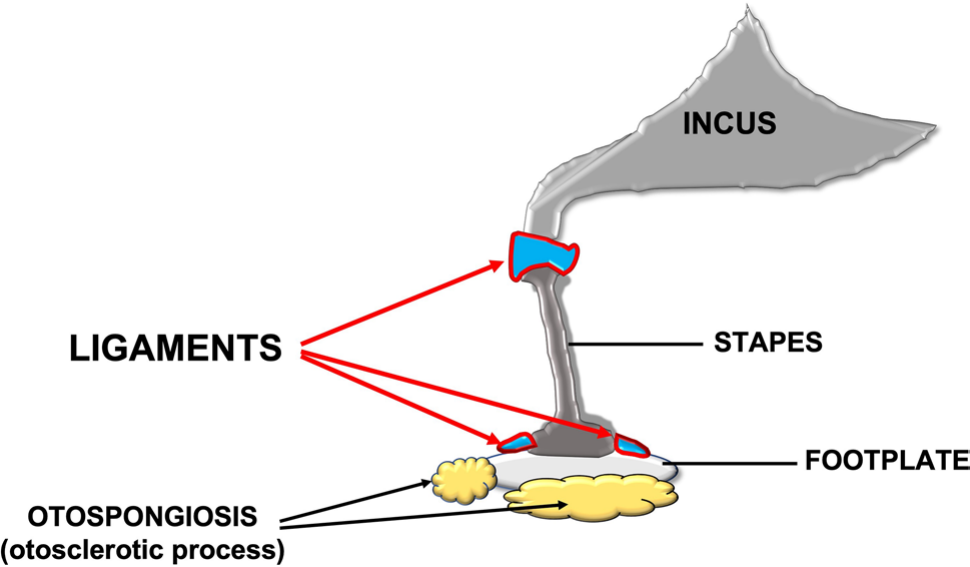
The ossicular chain and the ligaments involved in the mobility of it (red arrows). The black arrows show the areas where the otosclerotic process causes hypomobility or fixity of the footplate determining the conductive hearing loss that affects patients with otosclerosis

Otosclerosis arises in young women and often during or immediately after pregnancy by the forth decade of age; the hormone storm (pregnancy) or the hormonal change (decrease/increase) during women’s life, associated to a hereditary base, can be responsible for the disease onset [12, 13]. Otosclerosis is characterized by an imbalanced pathologic bone turnover that leads to a progressive fixation of the stapes footplate causing CHL [13]. Because female hormones are pro-inflammatory [14] and the inflammation increases the bone turnover [9], during pregnancy the estrogen increase causes the otosclerosis onset. The same inflammatory trigger could be observed in the presence of hormonal changes during menstrual cycle and aging [8, 9]. This (deleterious) estrogen effect has been confirmed by several clinical studies, in which most subjects affected by otosclerosis were women [15, 16]. Because of this strong epidemiologic effect and the biologic explanation of the phenomenon, few studies have been conducted to compare the hearing abilities in otosclerotic females and males [15, 17, 18]. Furthermore, none of the studies identified differences in auditory threshold and air bone gap (ABG) among sex. The ABG—the difference between air conduction and bone conduction audiometric thresholds [19]—is extremely important because it indicates how the sound is transmitted through the middle ear (movement of the ossicular chain), and indicates the severity of fixation of the stapes (Fig. 1) and may predict better operatory outcomes [20].

The ossicular chain contains some ligaments whose elasticity has an impact on the hearing abilities [21, 22]; we therefore speculate that the elasticity of these structures might have an impact on the ABG and the transmission of the sounds also in patients with otosclerosis.

Recent studies have shown that female hormones increase the concentration of collagen fiber making the women ligaments more elastic compared to men [23, 24]. These structures might be more elastic also in women with otosclerosis, determining smaller ABG and better auditory thresholds.

To investigate this hypothesis, we retrospectively analyzed the presence of differences in the hearing thresholds (air and bone conduction and ABG) before and after stapedotomy between females and males.

## Material and methods

A retrospective analysis of 184 medical charts (123 women, 61 men; age average 50.9 years + 12.1), for a total of 231 stapedoplasty (50 bilateral otosclerosis) was performed, including patients who underwent surgery between 2013 and 2020. This study was hosted at the Department of Head and Neck Surgery of the Silvestrini University Hospital, Perugia. The Internal Review Board of the hospital approved the projects without release of ID as expected by national law for retrospective studies and was conducted in accordance with the ethical standards of the Declaration of Helsinki. All patients included in this study signed a written consent and authorized the use of their data for research purpose.

We included patients who had on file pre- and post-operatory pure tone audiometry (PTA) and pre-operatory tympanometry and stapedial reflex test (SRT). These two tests were performed only before surgery to avoid the damage of the prosthesis (fluoroplastic piston). The PTA was performed pre-surgery (T0) 1 week after surgery (T7), two weeks (T15), and 1 month later (T30).

Pure tone Audiometry (PTA). The test was conducted using earphones in a silent cabin; both air and bone conduction thresholds were measured. To test the *air conduction* threshold, a pulse tone was emitted by the earphone on the side of the ear that had to be tested without masquerading sound. This procedure was performed bilaterally. The sound stimulation started from 10 dB, with increases of 10 dB and decreases of 5 dB, to confirm the individual sound perception at each frequency. The impulse for each frequency tested (250, 500, 1000, 2000, 4000 Hz) was sent three times, following the method described above. To test the *bone conduction,* an electromedical earphone was placed on the mastoid of the ear that had to be tested and the sound stimulation was performed exactly as for air thresholds. Also in this case, the test was performed bilaterally. ABG was calculated measuring the difference between air conduction and bone conduction audiometric thresholds [19].

Stapedial Reflex Test (SRT) was performed bilaterally. A probe was inserted in the tested ear and the reflex was evaluated at 500, 1000, 2000, and 4000 Hz. A continuous stimulus was sent starting with intensity 70 dB HL up to 105 dB HL in 5 dB steps until an acoustic reflex threshold is obtained.

The diagnosis of otosclerosis was based on normal otoscopic findings, progressive conductive hearing loss as air conduction (AC) pure tone average > 30 dB in the range of 0.5–4 kHz, and absence of stapedius reflexes.

Surgery was always performed by the same senior surgeon (GR) to reduce the operator’s related risk of bias. Stapedoplasty was always performed in the same condition under general anesthesia, by transcanalar approach to reach the stapes and using “cold” instruments (microperforator). The same prosthesis fluoroplastic piston of 0.4 mm diameter and 4.2 mm length (Richards Platinum Fluoroplastic Pistons by Olympus) was used in all cases.

Data on age, sex, type of surgery, side of surgery, and auditory (air and bone) thresholds pre- (T0) and one month after surgery (T30) were used.

For this study, we considered T0 and T30 because 30 days of follow-up allowed to identify homogeneous results after surgery, excluding the post-surgery inflammations which could negatively impact the auditory results.

### Statistical analysis

The prevalence of sex was calculated, as well as the prevalence of the side. As first, we compared the air threshold at T0 and T30 singularly in females and males by one-way ANOVA and same was done for bone thresholds. Bonferroni–Holm (BH) ad hoc was performed. ABG average at T0 and T30 was compared using double-tailed *t* test (τ). All patients were included independently from the side of affection. Secondly, we compared pre- (T0) and post-auditory thresholds (T30) (air and bone) of females and males by one-way ANOVA. Moreover, we also compared each frequency (500, 1000, 2000, and 4000 Hz) to identify specific differences. Thirdly, we compared the average ABG pre- and post-surgery among sex, and only in case of statistically significant differences, we compared the ABG at 500, 1000, 2000, and 4000 Hz. Because the samples have different sizes, the Cohen’s d coefficient was performed to evaluate the effect of this difference. We analyzed the sample effect looking at both pre- and post-average air conduction and bone conduction thresholds, difference between sex at 500,1000, 2000, and 4000 Hz (calculation only in case of statistically significant differences), ABG pre- and post-surgery, and difference in ABG. *P* was considered significant < 0.05. The statistical analyses were performed by Stata^®^.

## Results

### General results

Table 1 summarizes the general characteristics of our sample. The sample included 123 (66.8%) women and 61 (33.2%) men; all patients were white Caucasian. Patients included in this study were affected by otosclerosis for at least 2 years.

**Table 1:** Demographic characteristics of the sample

	Women	Men	
Gender	147	82	
Age	50 ± 11.57	52.5 ± 13.1	
**Site of hearing loss**	**Right**	**Left**	**Bilateral**
	62 women	55 women	30 women
	42 men	30 men	10 men

± Standard deviation

**Fig. 2:**
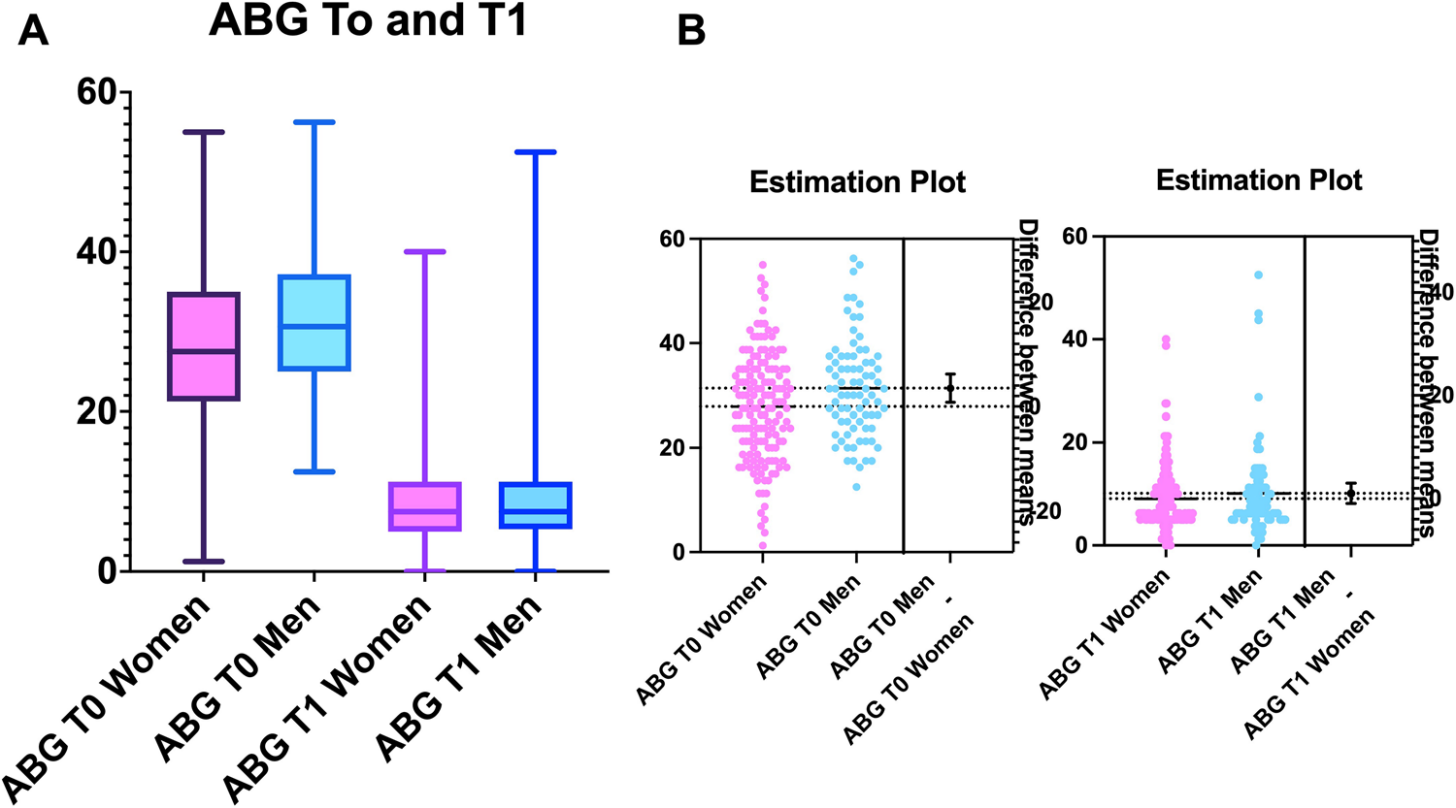
**A** The plot comparing the average air bone gap (ABG) among sex before and after surgery. The pink boxes represent the women’ values, the blue one’s men. Median and 95% confidence interval are reported. “Y axis” indicates the decibel (dB); a score over 15 dB is meaningful of hearing deficit. After surgery both women and men recovered normal auditory function

Females showed a prevalence of right CHL (61.7%). Men presented a slightly prevalence of right CHL (55.5%). All patients presented good bone conduction thresholds before and after surgery (Table 2) and equally recovered the air threshold and ABG after surgery (Fig. 2A, B).

**Table 2:** Air Conduction (AC) and Bone Conduction (BC) thresholds

	500 Hz	500 Hz	1000 Hz	1000 Hz	2000 Hz	2000 Hz	4000 Hz	4000 Hz
	BC T0	BC T30	BC T0	BC T30	BC T0	BC T30	BC T0	BC T30
Women	21.8 ± 10.3	19.9 ± 10.4	24.3 ± 11.6	21.25 ± 11.3	36.75 ± 15	29.5 ± 14.6	28.65 ± 15.7	30.85 ± 17.4
Men	21.6 ± 11	21.3 ± 11.3	25.9 ± 12.8	25.3 ± 13.7	39.3 ± 17.9	33.2 ± 19.5	32.9 ± 19.9	37.1 ± 19

	AC T0	AC T30	AC T0	AC T30	AC T0	AC T30	AC T0	AC T30
Women	57.9 ± 13.1	28.6 ± 13	57 ± 12.6	29.8 ± 14	52.1 ± 16	34 ± 15.6	53.7 ± 20.7	43.9 ± 20.7
Men	60 ± 14.6	31.1 ± 17.4	61.4 ± 16	34.8 ± 19.7	58.7 ± 21.1	38.4 ± 23.7	65.2 ± 27.1	51 ± 25.5

± Standard deviation

Table 2 shows the average of bone conduction (BC) and air conduction (AC) threshold in females and males before and after stapedoplasty.

### Comparison of audiologic data

#### Within gender

Females: the analysis of air conduction thresholds (averaging between right and left sides) showed statistically significant differences comparing the frequency at T0 and T30 (ANOVA: *p* < 0.0001); in particular, statistically significant differences before and after surgery were observed for 500 Hz (BH: *p* < 0.01), 1000 Hz (BH: *p* < 0.01), 2000 Hz (BH: *p* < 0.01), and 4000 Hz (BH: *p* < 0.01).

No statistically significant differences were observed comparing bone conduction thresholds (ANOVA: *p* > 0.05) before and after surgery for frequencies 500, 1000, 2000, and 4000 Hz; in fact, otosclerosis generally determines CHL. Only severe forms of the disease affect the cochlea causing SNHL [25]. ABG average comparing T0 and T30 showed statistically significant differences (τ: *p* < 0.0001).

Males: the frequential analysis of the air conduction thresholds (averaging between right and left sides) showed statistically significant differences comparing the frequency at T0 and T30 (ANOVA: *p* < 0.0001); in particular, statistically significant differences were observed for 500 Hz before and after surgery (BH: *p* < 0.01), 1000 Hz (BH: *p* < 0.01), 2000 Hz (BH: *p* < 0.01), and 4000 Hz (BH: *p* < 0.01). As in females, no statistically significant differences were observed comparing bone conduction thresholds before and after surgery (ANOVA: *p* > 0.05). ABG average comparing T0 and T30 showed statistically significant differences (τ: *p* < 0.0001).

#### Between gender

**Fig. 3:**
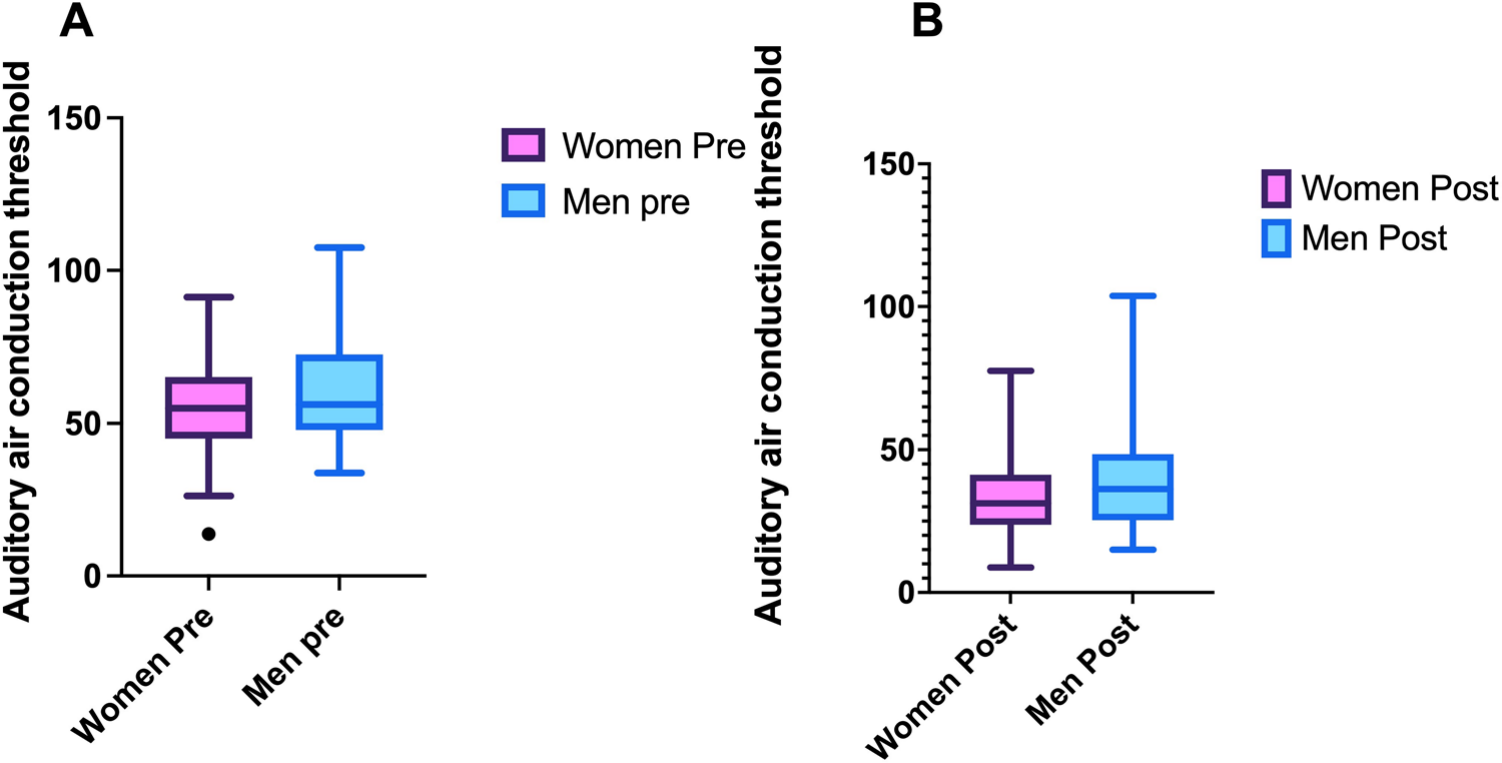
**A** The plots showing the differences in the air conduction thresholds (averaging 500–4000 Hz) before surgery and after surgery (**B**). “Y axis” indicates the decibel (dB). The point indicates the statically significant difference, which was clearly understandable also looking at the boxes, it is present before surgery but not after

T0 air conduction thresholds (averaging between right and left sides) comparing females and males showed no statistically significant differences (ANOVA: *p* > 0.05). However, specifically observing the singular frequency (500, 1000, 2000, and 4000), 4000 Hz presented statistically significant differences among gender (*p* < 0.01) (Fig. 3A).

**Fig. 4:**
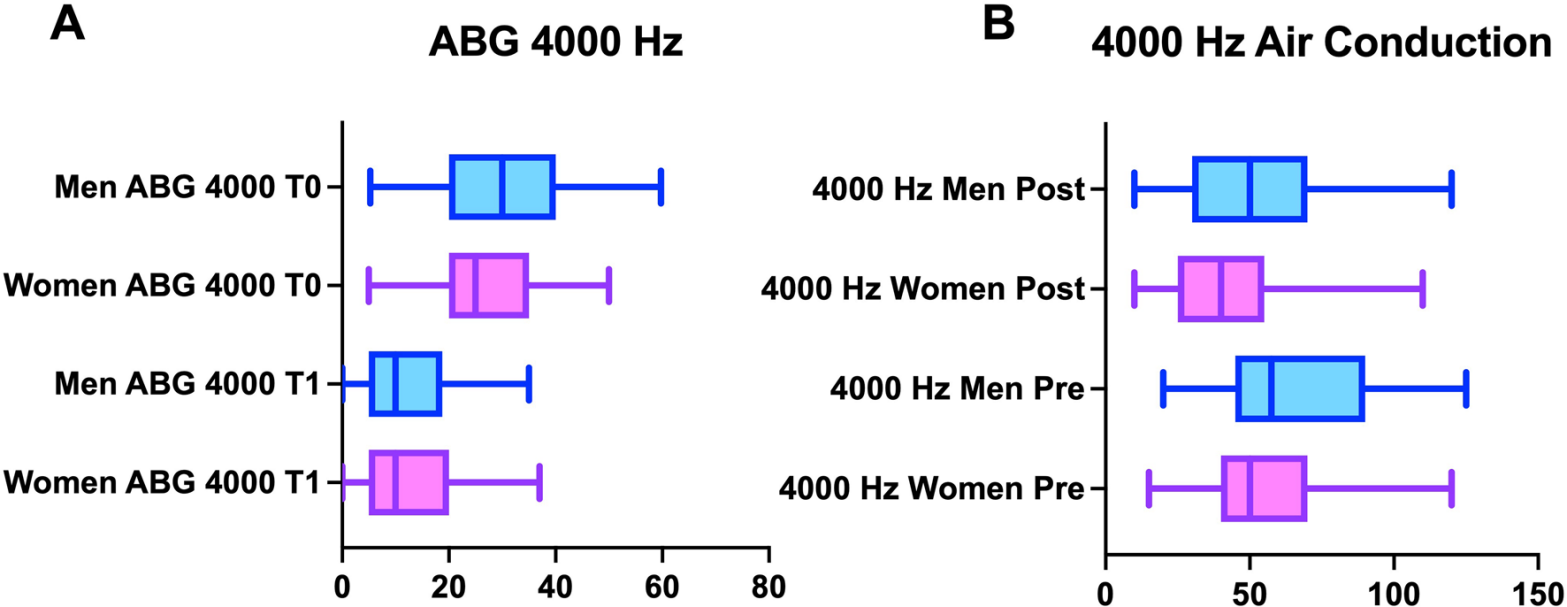
**A** The graph showing the differences in the ABG at 4000 Hz between women (pink) and men (blue); after surgery the difference is smaller but does not disappear. **B** The graph showing the differences in the air conduction threshold at 4000 Hz between women (pink) and men (blue); after surgery the difference among sex is smaller but still present

T30 air conduction thresholds (averaging between right and left sides) showed generally no statistically significant differences between women and men (ANOVA: *p* > 0.05). The analysis for singular frequency showed statistically significant differences at 4000 Hz (*p* < 0.05)—exactly as observed in the pre-operatory analysis (Figs. 3B, 4B).

No statistically differences were observed comparing bone conduction thresholds between women and men before and after surgery (ANOVA: *p* > 0.05).

The analysis of ABG average showed statistically significant differences between females and males (ANOVA: *p* < 0.0001); the differences in the ABG were identified comparing T0 and T30 among sex (Figs. 2A, B). The ABG average showed statistically significant differences at T0 between women and men (Women: 27.8; SD: 10.3 vs Men: 31.4 SD: 9.4; HB: *p* = 0.04). However, no statistically significant differences were observed after surgery (women: 9; SD: 6.3 vs men: 10.1 SD: 8.9; HB: *p* = 0.8).

Looking at ABG for specific frequencies (500, 1000, 2000, and 4000 Hz), the statistically significant difference identified at T0 between women and men (ANOVA: *p* < 0.0001) was found for 4000 Hz (HB: *p* < 0.05) (Fig. 4A).

Because the two groups had different sizes, we calculated the effect of the sample size as described in the statistical method section. The effect was small comparing average air conduction before (0.465; 95% CI 0.19–0.739) and after surgery (0.369; 95% CI 0.096–0.642) and air conduction thresholds at 4000 Hz pre-surgery (0.498; 95% CI 0.223–0.772). It was also small for the average bone conduction thresholds post-surgery (0.263; 95% CI -0.009–0.535), ABG average pre-surgery (0.356; CI95%:0.082–0.629), and ABG at 4000 Hz pre-surgery (0.337; CI95%: 0.065–0.61).

The Cohen’s effect was very small for the average bone conduction thresholds pre-surgery (0.174; CI95%: − 0.098 to 0.045), average ABG post-surgery (0.151; 95% CI − 0.121 to 0.422), and ABG at 4000 Hz post-surgery (0.098; 95% CI − 0.173 to 0.37).

## Discussion

The main finding of our study was that males presented worse high-frequency thresholds (4000 Hz) compared to females both before and after surgery; this difference was independent from age. These results might suggest that, independently from their blood concentration, female hormones might impact on the ligament elasticity improving the ossicular chain movements [21, 22]. Furthermore, we found that all the patients that preserved good bone conduction thresholds successfully recovered their auditory function, thus confirming the previous studies about the efficacy of surgery in these type of patients [15, 17, 18, 26, 27].

We also analyzed the ABG among males and females, and the results showed that also in this case females presented better thresholds than males. This result is opposite to other authors’ observations [17], in which differences among sex were not found. However, we think that, because the limited number of studies that focused on sex difference in patients with otosclerosis, only studies on larger samples can confirm (or disconfirm) our results.

To improve the power of our study, because of the different sample sizes, we performed Cohen’s test, which showed that the effect of the differences on our analysis varied from very small to small, confirming that our results were reliable despite the differences in the sample size.

Women preserved better auditory functions, although our database included patients in menopause; it seems that the effects of the female hormone on the ligaments of the ossicular chain were not impacted by aging. In fact, aging causes the reduction of ligaments elasticity both in males and females, but this change is always relative to the starting condition. Therefore women, who have more elastic ligaments, maintain this advantage also by aging. Moreover, the inner ear works better in females than in males thanks to the effect of female hormones since birth [28]**;** this effect persists despite menopause, because there never is a complete inversion of the hormone rate (testosterone > estrogen/progesterone).

Looking at the bone conduction thresholds only—which evaluate the function of the inner ear—we did not identify any differences. This data is in the opposite of other authors [3], who identified better preservation of high frequency (3000–6000 Hz) in women than in men in large samples of patients with SNHL. The main differences among Park’s study and ours was that the authors analyzed people affected by age-related SNHL and in this case the inner ear was affected by the disease. On the contrary, our study focused on the function of middle ear, making the results of the studies not comparable. Moreover, it is known that otosclerosis affects the inner ear and can modify the perilymph, thus altering the function of the HC [29, 30] and inducing a form of SNHL. We speculate that this negative effect might have reduced the benefit of female hormone (preservation of better inner ear functions [4]) and sloped down the AC threshold of the women. This reduction, even if not substantial and extremely pathologic, might explain the absence of differences in the AC thresholds among sex.

In our patients, we identified better conductive thresholds, because of the positive effect of the hormone on the middle ear ligaments (Fig. 1). At 4000 Hz, females had better conductive threshold and ABG pre-surgery. But why only this frequency was statistically different between females and males? The dynamic of sound transmission in the middle ear can be helpful to interpret this data [21, 22, 31]. High-frequency sounds generate [31] waves with high amplitude and short latency, which disperse from basal turn to the apex of the cochlea. This characteristic of high-frequency sounds requests that the ossicular structures have sufficient flexibility to sustain the mechanic stimulus; this capacity is guarantee by middle ear ligaments. This is the reason why even in the presence of the osteosclerotic process of the stapes footplate (Fig. 1) which reduce the movement on the oval window, preservation of good elasticity of the ligament allows a better transmission of sound as evidenced by small ABG and, better AC in the women.

Interestingly, the auditory differences in the ABG at 4000 Hz were equally present, despite smaller, after surgery (*p* < 0.01 at T0 and *p* < 0.05 at T30) and both sexes improved threshold after surgery (Figs. 4A, B). These data confirm that better ligaments elasticity (baseline ifference among sex) is beneficial even after the surgery (Fig. 1).

Our study suggests that the sex differences in the preservation the high-frequency hearing are not an exclusive prerogative of healthy population [3]. In osteosclerotic patients, although the disease is a woman’s prerogative, the female hormone represents the “angel and devil” effect. In fact, they predispose women to be affected from the disease more than men [16, 32], but thanks to the benefic effect of the hormones on the ligament [23, 24], the female population presented better auditory thresholds.

Our results could also explain the clinical evidence recently discovered by Macielack [33]; the authors analyzed 1169 women (case control study) and did not identify any relationship between endogenous estrogen exposure and development of otosclerosis, measured as worsening of the auditory function. Female hormones increase during pregnancy might improve the ligament elasticity and hide the auditory deficit linked to the progression of footplate fixation under hormonal influence. Additional sex studies are necessary to deeply clarify these aspects and confirm this theory. In particular, the future studies should be addressed both on clinic and temporal bone analysis. The first should evaluate women at different ages, affected by osteosclerotic diseases in early, middle, and late stage measuring the blood concentration of estrogen and progesterone. The temporal bone studies should be addressed in the comparison of ligament elasticity between female and male both in normal and osteosclerotic subjects.

Limitations of this study: this study presents several limitations. The first is the absence of measurement of the hormone’s levels, so the link between hormones and auditory thresholds is only based on the clinical evidence and therefore speculative. However, we are currently performing a prospective study that evaluates hormone concentration in the blood of the patients and their auditory thresholds to understand if a variation in hormone level can impact hearing recovery. Another important limitation was that we collected data on the frequencies between 500 and 4000 Hz only, and this did not allow understanding if there was an “estrogen effect” on the frequencies over or under the ones studied.

Based on the recent clinical evidence [33], the speculation of experimental studies [4], and the results of our study, we might conclude that female hormones in patients with otosclerosis are more beneficial than harmful; in fact, although surgery was equally beneficial in both men and women, the latter tended to preserve better auditory thresholds than men, despite being more affected by the disease.

## Conclusion

The results of our study support the hypothesis that female hormones could preserve the auditory capacities also in patients with otosclerosis. Despite women are affected by otosclerosis twice as men, their hormones positively impact the ossicular chain elasticity allowing to have better hearing thresholds compared to those of men affected by the same disease. Female hormones act on the ligaments that in females are more elastic than in males. The better elasticity allows to preserve the motility of ossicular chain even in the presence of otosclerosis of the footplate, thus determining the relative advantage we observe in women, with preservation of hearing abilities especially at high frequencies (4000 Hz). Additional studies on larger samples to analyze the effects of circulating estrogens on hearing capacity are necessary to confirm our results.

## Data Availability

The anonymized data are available under request of the corresponding author.

## References

[1] Shuster BZ, Depireux DA, Mong JA, Hertzano R (2019). Sex differences in hearing: Probing the role of estrogen signaling. J Acoust Soc Am.

[2] Barrero JP, López-Perea EM, Herrera S, Mariscal MA, García-Herrero S (2020). Assessment and modeling of the influence of age, gender, and family history of hearing problems on the probability of suffering hearing loss in the working population. Int J Environ Res Public Health.

[3] Park YH, Shin SH, Byun SW, Kim JY (2016). Age- and Gender-related mean hearing threshold in a highly-screened population: the Korean National Health and Nutrition Examination Survey 2010–2012. PLoS ONE.

[4] Delhez A, Lefebvre P, Péqueux C, Malgrange B, Delacroix L (2020). Auditory function and dysfunction: estrogen makes a difference. Cell Mol Life Sci.

[5] McFadden D (2009). Masculinization of the mammalian cochlea. Hear Res.

[6] McFadden D (1998). Sex differences in the auditory system. Dev Neuropsychol.

[7] Hultcrantz M, Simonoska R, Stenberg AE (2006). Estrogen and hearing: a summary of recent investigations. Acta Otolaryngol.

[8] McFadden D, Champlin CA, Pho MH, Pasanen EG, Maloney MM, Leshikar EM (2021). Auditory evoked potentials: differences by sex, race, and menstrual cycle and correlations with common psychoacoustical tasks. PLoS ONE.

[9] Lien KH, Yang CH (2021). Sex differences in the triad of acquired sensorineural hearing loss. Int J Mol Sci.

[10] Williamson TT, Ding B, Zhu X, Frisina RD (2019). Hormone replacement therapy attenuates hearing loss: mechanisms involving estrogen and the IGF-1 pathway. Aging Cell..

[11] Kim MT, Lee JH, Carpena NT, Lee MY, Chung PS, Jung JY (2021). Estrogen Replacement reduces hearing threshold shifts and cochlear hair cell loss after acoustic overexposure in ovariectomized rats. Clin Exp Otorhinolaryngol.

[12] Chole RA, Mckenna M (2001). Pathophysiology of otosclerosis. Otol Neurotol.

[13] Foster MF, Backous DD (2018). Clinical evaluation of the patient with otosclerosis. Otolaryngol Clin N Am.

[14] Schmidt M, Naumann H, Weidler C, Schellenberg M, Anders S, Straub RH (2006). Inflammation and sex hormone metabolism. Ann N Y Acad Sci.

[15] Sakihara Y, Parving A (1999). Clinical otosclerosis, prevalence estimates and spontaneous progress. Acta Otolaryngol.

[16] Crompton M, Cadge BA, Ziff JL, Mowat AJ, Nash R, Lavy JA, Powell HRF, Aldren CP, Saeed SR, Dawson SJ (2019). The epidemiology of otosclerosis in a British cohort. Otol Neurotol.

[17] Vartiainen E (1999). Sex differences in patients with hearing impairments caused by otosclerosis. Eur Arch Otorhinolaryngol.

[18] Kishimoto M, Ueda H, Uchida Y, Sone M (2015). Factors affecting postoperative outcome in otosclerosis patients: predictive role of audiological and clinical features. Auris Nasus Larynx.

[19] Scarpa A, Ralli M, Cassandro C, Gioacchini FM, Greco A, Di Stadio A, Cavaliere M, Troisi D, de Vincentiis M, Cassandro E (2020). Inner-ear disorders presenting with air-bone gaps: a review. J Int Adv Otol.

[20] Peñaranda D, Moreno S, Montes F, Garcia JM, Rico Z, Peñaranda A (2021). Fifteen-year follow-up of stapedotomy patients: audiological outcomes and associated factors in a middle income country. Audiol Neurootol.

[21] Chen SI, Lee MH, Yao CM, Chen PR, Chou YF, Liu TC, Song YL, Lee CF (2013). Modeling sound transmission of human middle ear and its clinical applications using finite element analysis. Kaohsiung J Med Sci.

[22] Ramier A, Cheng JT, Ravicz ME, Rosowski JJ, Yun SH (2018). Mapping the phase and amplitude of ossicular chain motion using sound-synchronous optical coherence vibrography. Biomed Opt Express.

[23] Leblanc DR, Schneider M, Angele P, Vollmer G, Docheva D (2017). The effect of estrogen on tendon and ligament metabolism and function. J Steroid Biochem Mol Biol.

[24] Hansen M (2018). Female hormones: do they influence muscle and tendon protein metabolism?. Proc Nutr Soc.

[25] Messineo D, Ralli M, Greco A, Di Stadio A (2021). Double ring in cochlear otosclerosis: a limit to cochlear implantation? The solution is the surgical approach. Ear Nose Throat J..

[26] Salmon C, Barriat S, Demanez L, Magis D, Lefebvre P (2015). Audiometric results after stapedotomy operations in patients with otosclerosis and preoperative small air-bone gaps. Audiol Neurootol..

[27] Blijleven EE, Wegner I, Tange RA, Thomeer HGXM (2019). Revision stapes surgery in a tertiary referral center: surgical and audiometric outcomes. Ann Otol Rhinol Laryngol..

[28] Burke SM, van Heesewijk JO, Menks WM, Klink DT, Kreukels BPC, Cohen-Kettenis PT, Bakker J (2020). Postnatal effects of sex hormones on click-evoked otoacoustic emissions: a study of adolescents with gender dysphoria. Arch Sex Behav.

[29] Chevance LG, Causse J, Bretlau P, Jorgensen MB, Bergés J (1972). Hydrolytic activity of the perilymph in otosclerosis. A preliminary report. Acta Otolaryngol..

[30] Silverstein H, Schuknecht HF (1966). Biochemical studies of inner ear fluid in man. Changes in otosclerosis, Meniere’s disease, and acoustic neuroma. Arch Otolaryngol..

[31] Di Stadio A (2017). Which factors to induce hearing loss in professional musicians? Extensive literature review and histopathology findings can answer it. Hear Balance Commun.

[32] Gristwood RE, Venables WN (1983). Pregnancy and otosclerosis. Clin Otolaryngol Allied Sci.

[33] Macielak RJ, Marinelli JP, Totten DJ, Lohse CM, Grossardt BR, Carlson ML (2020). Pregnancy, estrogen exposure, and the development of otosclerosis: a case-control study of 1196 women. Otolaryngol Head Neck Surg.

[34] Della Volpe A, Ippolito V, Roccamatisi D, Garofalo S, De Lucia A, Gambacorta V, Longari F, Ricci G, Di Stadio A (2020). Does unilateral hearing loss impair working memory? An Italian Clinical study comparing patients with and without hearing aids. Front Neurosci.

